# District health manager and mid-level provider perceptions of practice environments in acute obstetric settings in Tanzania: a mixed-method study

**DOI:** 10.1186/s12960-016-0144-5

**Published:** 2016-08-08

**Authors:** Njoki Ng’ang’a, Mary Woods Byrne, Margaret E. Kruk, Aloisia Shemdoe, Helen de Pinho

**Affiliations:** 1 Center for Children & Families, School of Nursing, Columbia University, 617 West 168th Street, Georgian Building Room 346, New York, NY United States of America; 2 Department of Health Policy and Management, Mailman School of Public Health, Columbia University, New York, NY United States of America; 3 Ifakara Health Institute, Dar es Salaam, Tanzania; 4 Averting Maternal Death and Disability Program (AMDD), Heilbrunn Department of Population and Family Health, Mailman School of Public Health, Columbia University, New York, NY United States of America

**Keywords:** Council health management teams, Emergency obstetric care, Human resources management, Mid-level providers, Practice environment, Tanzania

## Abstract

**Background:**

In sub-Saharan Africa, the capacity of human resources for health (HRH) managers to create positive practice environments that enable motivated, productive, and high-performing HRH is weak. We implemented a unique approach to examining HRH management practices by comparing perspectives offered by mid-level providers (MLPs) of emergency obstetric care (EmOC) in Tanzania to those presented by local health authorities, known as council health management teams (CHMTs).

**Methods:**

This study was guided by the basic strategic human resources management (SHRM) component model. A convergent mixed-method design was utilized to assess qualitative and quantitative data from the *Health Systems Strengthening for Equity: The Power and Potential of Mid*-*Level Providers* project. Survey data was obtained from 837 mid-level providers, 83 of whom participated in a critical incident interview whose aim was to elicit negative events in the practice environment that induced intention to leave their job. HRH management practices were assessed quantitatively in 48 districts with 37 members of CHMTs participating in semi-structured interviews.

**Results:**

The eight human resources management practices enumerated in the basic SHRM component model were implemented unevenly. On the one hand, members of CHMTs and mid-level providers agreed that there were severe shortages of health workers, deficient salaries, and an overwhelming workload. On the other hand, members of CHMTs and mid-level providers differed in their perspectives on rewards and allocation of opportunities for in-service training. Although written standards of performance and supervision requirements were available in most districts, they did not reflect actual duties. Members of CHMTs reported high levels of autonomy in key HRH management practices, but mid-level providers disputed the degree to which the real situation on the ground was factored into job-related decision-making by CHMTs.

**Conclusions:**

The incongruence in perspectives offered by members of CHMTs and mid-level providers points to deficient HRH management practices, which contribute to poor practice environments in acute obstetric settings in Tanzania. Our findings indicate that members of CHMTs require additional support to adequately fulfill their HRH management role. Further research conducted in low-income countries is necessary to determine the appropriate package of interventions required to strengthen the capacity of members of CHMTs.

## Background

Poor practice environments alternately induce and exacerbate low levels of health worker performance in sub-Saharan Africa, thus undermining delivery of maternal health services needed to reduce the maternal mortality burden in the region. Women lack access to skilled providers when personnel shun service in poor practice environments. Poor practice environments are characterized by inadequate compensation and supervision, lack of basic equipment and supplies, and ill-maintained facilities [1–7]. Negative provider attitudes and disrespectful treatment of patients in poor practice environments compromise quality of care and deter women from seeking safer facility births in areas where maternal mortality is high [8, 9]. As a result, researchers and policymakers alike are turning their attention to the role played by practice environments in shaping the performance of personnel who deliver maternal health services in sub-Saharan Africa.

According to the International Collaborating Partners of the Positive Practice Environment Campaign^1^, positive practice environments are those which enable a motivated, productive, and high-performing pool of personnel by (1) recognizing their professional autonomy, (2) rewarding employee performance, (3) employing effective management practices, (4) offering opportunities for professional development, (5) adopting safety standards, and (6) ensuring the well-being of personnel [10]. These ideal healthy, supportive, and safe work environments are difficult for health personnel to attain regardless of profession, practice setting, and high- or low-income country status [11–13]. For a select group of facilities in the United States (US) known to epitomize positive practice environments, personnel demonstrate high levels of performance corresponding to a superior quality of health services. Studies of these outliers single out managers who endorse positive and enabling practice environments as the essential component of their success [14, 15]. The leaders exhibit four key attributes: (1) a deep commitment to an organizational culture centered on quality, (2) ability to attract and retain the right talent to accomplish quality-driven goals, (3) implementing appropriate processes for quality improvement, and (4) providing “staff with the right tools to do their job” [14, 15].

In sub-Saharan Africa, the capacity of human resources for health (HRH) managers to strategically plan for and deploy health personnel is weak. In 26 countries assessed, HRH managers were found to lack adequate infrastructure, training, and work experience to fulfill even basic management activities [16]. The ability of personnel to respond to emerging maternal health needs is compromised when critical elements of practice environments dependent upon the direct action of HRH managers are absent [14, 15, 17]. We propose that HRH managers are vitally important to high-quality maternal health care and in turn a relevant part of the post-Millennium Development Goal (MDG) agenda calling for the global community to renew its commitment to maternal health [18–20].

As in other sub-Saharan African countries, the HRH management capacity in the United Republic of Tanzania is considered weak. The decentralized health system in Tanzania grants local (district) authorities, known as council health management teams (CHMTs)^2^, the mandate to plan for, implement, and regularly monitor the delivery of health services [21, 22]. National supportive supervision guidelines only require members of CHMTs to conduct supervisory visits in health facilities quarterly [23]. A study evaluating HRH management capabilities in the country concluded that they were fragmented at best [24]. The turnover rate for HRH managers, including CHMTs, was reported to be 50 %. Half of the HRH managers failed to meet government criteria that they are a health professional with additional qualification in public health. At least 42 % of HRH managers lacked a formal job description, which essentially rules out the possibility of evaluating their performance. At the national level, the HRH unit housed within the Ministry of Health that bears responsibility for overseeing HRH management activities was found to be poorly staffed [24].

Simultaneously, maternal mortality in Tanzania is high with 398 deaths per 100 000 live births; maternal mortality contributes significantly to the national burden of disease and is designated a priority health area [25]. Critical shortages of HRH in Tanzania mean the personnel vacancy rate in the last decade has been as high as 65 % [22]. With only 4.7 physicians, nurses, and midwives per 10 000 people, the availability of HRH is far below the recommended 23 physicians, nurses, and midwives per 10 000 people necessary to ensure 80 % coverage by skilled attendants during childbirth [26]. Consequently, only 50 % of deliveries in the country are supervised by a skilled provider [27]. Similar rates of skilled attendance at birth are observed in the sub-Saharan African region [27].

**Fig. 1:**
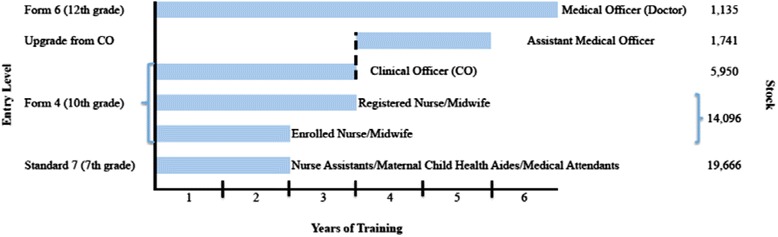
Differences in entry level, years of training, and stock of MLPs [31, 85, 86]

Tanzania’s response to its dual crises, high maternal mortality and scarce HRH, has been to train and license mid-level providers (MLPs) to perform an expanded set of basic and comprehensive emergency obstetric care (EmOC)^3^ services [28, 29]. MLPs typically possess 2–5 years of post-secondary education, which is less than 6 years of preservice training reserved for physicians, and include the following cadres: nurses, midwives, and associate clinicians (assistant medical officers [AMOs] and clinical officers [COs]) [28, 30, 31]. The shortage of MLPs and other trained personnel means that in practice, providers holding only a primary school education and little or no professional training (medical attendants, maternal child health aides, and nurse assistants) also conduct deliveries. For the purpose of our study, these three cadres with little or no professional training are considered one group identified as unlicensed assistive personnel (UAP). Differences in entry level, years of training, and stock of MLPs are depicted in Fig. 1. Beyond job titles and professional qualifications, there are discrepancies between training, licensure, and actual performance of basic and comprehensive EmOC functions by MLPs in Tanzania. For example, while MLPs are trained in a wide range of procedures, national and district policies limit their scope of practice to a smaller set [28].

Acute obstetric care is delivered in clinical settings typically characterized by unpredictable fluctuations in patient volume and acuity by a wide range of providers [32]. In order for these teams of providers to mobilize and deliver care that is reliable and safe, they must successfully navigate complex and dynamic organizational systems (structures, processes, and values) within the practice environment [33, 34]. Studies conducted in high-resource settings show that optimal work environments have in place systems which prioritize enhanced surveillance of patients by increasing availability of qualified providers at the bedside and supporting their decision-making capacity [11, 35]. There is evidence of growing interest to empirically examine the same concepts in low-resource milieu.

One such study conducted by McAuliffe and colleagues found that nursing and medical staff in Malawi responding to the Healthcare Provider Work Index (HPWI) rated their practice environments poorly largely due to weak supervision and inadequate resources [36]. Demonstrated in the McAuliffe paper is the empirical and conceptual utility of HPWI in identifying the theoretical units specific to ideal models of professional practice that have been observed in exemplary facilities, such as sufficient frontline staff members to provide optimal patient surveillance and collegial interdisciplinary relationships. Where composite measures like the HPWI perform less well is in describing work environments whose bureaucratic models are characterized by a hierarchical, transactional relationship between HRH managers and providers [35]. Our study aimed to build on already established methods of measuring perceptions of practice environments by departing from the professional practice model viewpoint to focus on the bureaucratic dimension. Specifically, we examined HRH management practices that are fundamental determinants of the quality of practice environments and the level of performance exhibited by health providers [14, 15, 37].

Studies conducted in Tanzania to examine HRH management practices have produced conflicting results. Members of CHMTs interviewed as part of the multi-country, multi-site mixed-method study *Health Systems Strengthening for Equity: The Power and Potential of Mid*-*Level Providers* (HSSE) collectively endorsed utilizing supervisory mechanisms to create supportive practice environments centered on helping health workers achieve their patient care goals [38]. Yet, personnel in rural southern Tanzania described persistence of poor practice environments characterized by inadequate resources and low motivation [39]. To better understand the paradox between HRH management practices utilized by members of CHMTs in Tanzania and persistence of poor practice environments, we explored data from the HSSE study. Like two sides of one coin, we assessed HRH management practices reported by members of CHMTs and gauged for concordance in perceptions reported by MLPs.

## Methods

### Theoretical framework

**Fig. 2:**
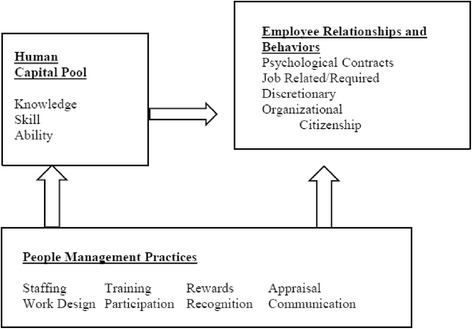
Basic strategic human resources management component model

Our understanding of the HRH management practices that contribute to the practice environment was guided by the theoretical underpinnings of Wright, Dunford, and Snell’s basic strategic human resources management (SHRM) component model (see Fig. 2) [40, 41]. The framework proposes that eight specific HRH management practices can be employed to assemble and deploy a pool of personnel who exhibit optimal knowledge, skills, attitudes, relationships, and behaviors that enhance organizational performance [40, 41]. These are staffing, work design, training, rewards, recognition, appraisal, communication, and participation.

### Design

We completed an analysis of data from the HSSE study utilizing a descriptive convergent mixed-method approach. The HSSE study is described in detail elsewhere [28, 38, 42]. In brief, the study aimed to generate evidence on the role of MLPs in maternal health service delivery in sub-Saharan Africa by collecting in parallel interview and survey data from local health authorities and MLPs in Malawi, Tanzania, and Mozambique [28, 38]. We used data from Tanzania. Core qualitative (interviews) and supplemental quantitative (surveys) components were merged to answer the research question [42]. The triangulation of methods characteristic of this design was done to facilitate synthesis, comparison, and corroboration of responses made by members of CHMTs and MLPs [43].

### Sample

Procedures used to select facilities from which participants in the HSSE study were recruited have been described elsewhere [42]. MLPs were identified by in-country research team members at their workplace and recruited following a purposeful sampling strategy in hospitals, health centers, and dispensaries across eight regions in Tanzania (Table 1). Purposeful sampling is used when the research goals demand participants who are knowledgeable in the phenomenon of interest to inform the research question [44, 45]. MLPs were eligible if they had performed one or more signal EmOC functions in the 3 months prior to meeting with the research team. Eligible members of CHMTs overseeing the districts from which we recruited MLPs were identified through pertinent policy literature, local research team members, and snowballing techniques. Recruitment of members of CHMTs and MLPs proceeded in snowball fashion. Snowballing in qualitative research is valuable in generating the maximum variation of responses; therefore, quality not quantity of responses is stressed to generate as rich an image as possible of the work environment [44–46].

**Table 1:** Demographic characteristics of MLPs responding to the Provider Survey

Demographic characteristics	Number (%)	Cadre	Number (%)
Female	627 (74)	Enrolled nurses	5 (0.6)
Age 26–55 years	716 (85)	Registered nurses	150 (18)
Full-time employment	717 (85)	Enrolled midwife	247 (30)
Permanent employment status	804 (95)	Registered midwife	20 (2)
Employed in government facility	744 (88)	Enrolled public health nurse	58 (7)
Highest level of training is certificate/diploma	469 (55)/230 (27)	Registered public health nurse	11 (1.3)
Highest level of basic education is standard 7/form 4	210 (25)/ 547 (65)	UAP	179 (21)
Mean length of time at facility	8.2 years	Clinical officer	99 (12)
Mean tenure as a health worker	15.4 years	Assistant medical officer	68 (8)

Mid-level providers recruited from 8 regions: Mbeya (23 %), Iringa (15 %), Mwanza (13 %), Tanga (13 %), Pwani (12 %), Dodoma (11 %), Mtwara (7 %), Tabora (6 %)

### Data collection

Once necessary ethical approvals were obtained, instruments developed by the HSSE study team based on previously validated instruments were used to gather interview and survey data during October and November of 2008. The research team obtained information from members of CHMTs to complete the 32-item HRH Information Survey, comprising dichotomous, nominal, and Likert-type questions. MLPs completed the Provider Survey, a 298-item questionnaire comprising dichotomous, nominal, and Likert-type questions, plus 15 items that were part of a discrete choice experiment. The CHMT HRH Information Survey and the Provider Survey encompassed the quantitative branch of the study. In the qualitative arm, trained interviewers conducted semi-structured interviews using the CHMT Interview Guide and the Provider Critical Incident Analysis instrument. MLPs participating in the critical incident interview were asked to identify and elaborate on a work-related event causing loss of motivation and inducing intention to leave in the 3 months prior to encounter with the research team. Trained interviewers met with members of CHMTs at predetermined locales and times. MLPs were usually interviewed at their place of work although scheduling preferences were accommodated. Following informed consent, participants were enrolled in the study. The interviews were conducted in Swahili, audio recorded, and transcribed verbatim. Transcripts were then translated into English in Microsoft Word (Microsoft, Washington, USA) prior to analysis.

### Data analysis

#### Qualitative data analysis

Interview data were systematically compressed or coded into categories from which an inference could be made using conventional content analysis [47–49]. Two researchers proficient in qualitative methods (NN, MWB) worked closely together and read the transcripts iteratively to achieve immersion. Codes or verbatim statements made by respondents representing concepts were extracted and then sorted into related categories. In keeping with qualitative research tradition, the following measures to ensure rigor were applied: (1) trustworthiness by adhering to data analysis procedures, (2) credibility by holding peer-debriefing sessions, (3) dependability by triangulating methods, (4) confirmability by maintaining an audit trail of detailed records, and (5) transferability through dissemination of results [46]. Interview data were analyzed using NVivo qualitative analysis software version 10 (QSR International, Victoria, Australia).

#### Quantitative data analysis

A summary of the demographic characteristics of members of CHMTs and MLPs was obtained. Descriptive statistics were calculated as follows. Proportions of MLPs and members of CHMTs endorsing various phenomena of interest were calculated. Chi-square tests were conducted to examine differences in categorical responses between cadres [50]. The Kruskal-Wallis test was used to evaluate differences in nonparametric ordinal responses between cadres [51]. The nine discrete MLP cadres displayed in Table 1 were collapsed into five (AMO, CO, nurses, midwives, and UAP) to avoid violating statistical assumptions of the Kruskal-Wallis test, which requires at least five cases per group, and to permit greater generalizability [52]. Cadres whose responses differed significantly were identified using the Mann-Whitney *U* test with the Bonferroni correction applied to account for multiple comparisons [51]. Statistical significance was set at *P* < 0.05. Data were analyzed using SPSS version 18 (IBM, New York, USA).

### Human subject considerations

The HSSE study was approved by the institutional review boards (IRBs) at Columbia University in New York and Ifakara Health Institute in Dar es Salaam, Tanzania.

## Results

### Demographic characteristics

#### Members of CHMTs

The 37 members of CHMTs comprised 13 reproductive and child health coordinators (35 %), 10 district medical officers (27 %), 11 district health secretaries (30 %), 1 nursing officer (3 %), and 2 whose roles were not identified. Fifty-eight percent of members of CHMTs reported they were trained in HRH management with most indicating that their HRH management training lasted from a few days to a few weeks. Two possessed postgraduate degrees in public health and public administration.

#### Mid-level providers

The Provider Surveys were completed by 847 participants. Excluded from the analysis were eight physicians and two participants who did not identify their profession. Demographic characteristics of MLPs responding to the Provider Survey are summarized in Table 1. Majority of MLPs were female (74 %), between 26 and 55 years of age (85 %), employed full time (85 %), in permanent positions (95 %), and in government facilities (88 %). The highest level of basic education attained by 65 % of MLPs was equivalent to the 10th grade. Fifty-five percent possessed a post-secondary certificate. Mean tenure at current facility and mean length of time as a health worker was 8.2 and 15.4 years, respectively. Out of 837 MLPs, 83 were randomly selected to participate in the critical incident interview. Of these, 57 % were female, 25 % male, and 18 % did not assign their gender. They identified their occupations as follows: nurses (42 %), midwives (32 %), COs (13 %), UAP (8 %), and AMOs (5 %).

### Quantitative component

#### Staffing

According to members of CHMTs, the total number of staff employed in each district was below levels recommended for individual cadres at each type of facility (Table 2). Similarly, 62 % of MLPs disagreed with the item “enough staff members to provide quality care” and 57 % disagreed with the item “enough staff to get the work done.” Midwives expressed higher levels of disagreement than AMOs (Table 3).

**Table 2:** Districts reporting adequate staffing levels and availability of written standards of performance and written supervision requirements

Cadre^a^	Staffing^b^			Written standards of performance^b^	Written supervision requirements^b^
	Dispensaries	Health Centers	Hospitals		
Assistant medical officer	N/A (22)^c^	19 % (32)	20 % (32)	73 % (45)	70 % (46)
Clinical officer	6 % (34)	19 % (31)	35 % (31)	72 % (46)	83 % (46)
Registered nurse/nurse midwife	64 % (25)	22 % (32)	37 % (32)	72 % (46)	83 % (46)
Enrolled nurse/nurse midwife	14 % (29)	13 % (30)	19 % (26)	72 % (46)	80 % (46)
MCH aid	86 % (21)	71 % (21)	91 % (21)	71 % (44)	74 % (42)
Medical attendant	42 % (33)	53 % (30)	55 % (29)	67 % (43)	71 % (45)

Data on nursing assistants not collected

^a^Data on MCH aids and medical attendants reported separately

^b^Figures in parenthesis represent the number of districts (out of 48) with data provided

^c^Assistant medical officers typically do not function in dispensaries

**Table 3:** Comparison of MLP responses to items in the Provider Survey

Items	Kruskal-Wallis *χ* ^2^	*P*	Cadre		*P*
			Mann-Whitney *U* (mean rank)		
Enough staff to get the work done	12.968	0.01	Midwives (167.45)	AMO (127.95)	0.001
My work schedule is fair	17.305	0.002	AMO (167.43)	Nurses (132.00)	0.001
			AMO (189.49)	Midwives (148.81)	0.001
			AMO (90.06)	CO (73.40)	0.015
			UAP^b^ (205.51)	Nurses (179.91)	0.016
			UAP (225.69)	Midwives (196.32)	0.009
I feel burned out from my work	32.573	0.000	Nurses (139.75)	AMO (108.37)	0.004
			Nurses (161.39)	CO (116.52)	0.000
			Nurses (190.32)	UAP (160.26)	0.005
			Midwives (156.61)	AMO (119.55)	0.002
			Midwives (178.53)	CO (126.31)	0.000
			Midwives (208.23)	UAP (171.75)	0.001
Manager makes sure that all staff concerns are heard before job decisions are made	20.069	0.000	UAP (212.63)	Nurses (175.26)	0.001
			UAP (239.81)	Midwives (191.34)	0.000
To make job decisions, my manager collects accurate and correct information	20.371	0.000	UAP (122.33)	AMO (96.09)	0.004
			UAP (214.86)	Nurses (173.74)	0.000
			UAP (237.08)	Midwives (190.61)	0.000
Manager clarifies decisions and provides additional information when requested by staff	17.415	0.002	UAP (213.09)	Nurses (175.87)	0.000
			UAP (236.42)	Midwives (192.66)	0.000
My manager offers adequate justification for decisions about my job	10.192	0.037	UAP (207.74)	Nurses (177.13)	0.005
Overall, the rewards I receive are fair	16.657	0.002	UAP (206.42)	Nurses (176.53)	0.005
			UAP (232.50)	Midwives (190.20)	0.000
I am satisfied with this supervision system	12.783	0.012	UAP (229.81)	Midwives (190.77)	0.001
			UAP (207.56)	Nurses (179.16)	0.009
I think this is a fair supervision system	13.434	0.009	AMO (174.23)	Midwives (149.32)	0.036
			CO (185.93)	Midwives (164.43)	0.057
			UAP (228.05)	Midwives (190.16)	0.001
			UAP (205.89)	Nurses (183.23)	0.037

#### Work design

Members of CHMTs reported that procedures to match workload to staffing numbers were available in 68 % of the 48 districts. Equal proportions of MLPs agreed or disagreed their workload was fair (39 and 40.5 %, respectively) with no difference noted across cadres (*χ*^2^ = 7.650, *P* = 0.105). While 52 % of MLPs endorsed working additional unscheduled hours to help out during busy periods or when understaffed, 62 % considered their work schedules fair. AMOs perceived more fair work schedules than COs, nurses, and midwives while UAP reported more fair work schedules than nurses and midwives (Table 3). COs, nurses, and midwives were perceived by members of CHMTs in 80 % of the districts to bear the greatest workload. Nurses and midwives reported higher levels of burnout than AMOs, COs, and UAP (Table 3). Forty-nine percent of MLPs reported feeling burned out at least once a month and 38 % felt burned out more than once a week.

#### Training

Members of CHMTs reported 96 % of 43 districts had formal plans to provide in-service training, and available opportunities were granted to MLPs based on individual performance evaluations (56 %), training needs (37 %), and unspecified criteria (3.7 %). However, 41 % of MLPs reported that opportunities for in-service training were offered by managers using unspecified criteria, and 41 % reported they were based on performance reviews (Table 4). Half of MLPs were satisfied with systems used to assign staff for in-service training (*χ*^2^ = 8.308, *P* = 0.081), and 55 % agreed the systems were fair (*χ*^2^ = 7.295, *P* = 0.121) with no differences across cadres.

**Table 4:** MLP responses to items on supervision and access to in-service training

Item	Nurses^a^ (%)	Midwives (%)	UAP (%)	CO^b^ (%)	AMO^b^ (%)
Supervision					
Formal supervision process with regular prearranged supervision	60	50	54	55	51
Supervision available if I request it from my line manager	4	7	4	3	9
Supervision consists of negative feedback when performance is poor	19	19	25	14	20
I never receive any supervision or feedback on my performance	20	24	17	26	17
Access to in-service training					
Offered by supervisor/manager	40	44	36	50	24
Based on a formal review of training need of each employee	17	20	23	12	12
Based on performance review focusing on skills required for the job	43	37	41	38	64

^a^Totals greater than 100 % due to selection of more than one response by some participants

^b^Totals less than 100 % due to missing data

#### Participation

In the 48 districts, members of CHMTs reported moderate to high levels of autonomy in performing these HRH management functions: determining staffing levels (77.1 %), personnel recruitment (81.3 %), determining access to in-service training (81.4 %), supervision and performance management (89.6 %), staff discipline (85.5 %), and dismissals (56.2 %). Moderate to low levels of autonomy were reported for setting salaries (64.6 %) and allowances (60 %). Fifty-six percent of MLPs did not think members of CHMTs made sure all staff concerns are heard before making decisions. UAP had higher levels of agreement than nurses and midwives (Table 3). Half of MLPs agreed that members of CHMTs collect accurate and correct information to make job decisions. UAP reported higher levels of agreement than AMOs, nurses, and midwives (Table 3). Fifty-six percent of MLPs agreed that members of CHMTs clarified decisions and provided additional information upon request. UAP reported higher levels of agreement than nurses and midwives (Table 3). Half of MLPs agreed that members of CHMTs offered adequate justification for job decisions. UAP reported higher levels of agreement than nurses (Table 3).

#### Rewards and recognition

Members of CHMTs reported mechanisms for rewarding or sanctioning personnel for their performance were used in 96 % of the 48 districts. Free uniforms (85 %), access to training (88 %), and allowances (90 %) were the most commonly used rewards. Eighty percent of the MLPs disagreed that rewards received were fair. UAP perceived significantly less fair rewards than nurses and midwives (Table 3). While 65 % of MLPs were satisfied with recognition received for their work, 79.6 % considered their pay inadequate. Late payments were received by 27 % of MLPs, and responses did not differ between cadres (*χ*^2^ = 21.044, *P* = 0.518). Partial salaries were received by 30.7 % of MLPs, 47 % of whom received less than half their normal salary.

#### Appraisal

Members of CHMTs reported that written supervision requirements were available for each cadre in 70 % of districts (Table 2). Formal supervision during regular prearranged meetings was encountered by 53 % of MLPs (Table 4), and 69 % perceived adequate supervision. UAP were more satisfied with their supervision and perceived more fair supervision than nurses and midwives whereas midwives perceived a less fair supervision system than AMOs and COs (Table 3).

#### Communication

In more than 67 % of districts, members of CHMTs reported communicating written standards of performance to MLPs (Table 2). Sixty-eight percent of MLPs reported that they have a written job description with no difference between cadres (*χ*^2^ = 45.45, *P* = 0.07). Written job descriptions adequately described the duties performed by 61 % of MLPs, but 48 % were asked to perform duties not in their job description several times a month.

### Qualitative component

#### Mid-level providers

Data from 83 interviews of MLPs were compressed into thematic and referential units forming the initial coding scheme of 35 codes labeled with verbatim statements contained in participant narratives. Related codes were grouped together to form five categories or themes. Agreement between codes generated by both researchers was 98 %.

##### Theme I: The work environment is so difficult it affects us psychologically; sometimes, we think of leaving, but we stay

Excessive patient volume created difficult work environments for under-resourced and understaffed MLPs who mentioned extending themselves to fulfill the professional obligation to meet patients’ needs. One nurse midwife reported “…you know when a woman comes for delivery you have to find a way to help her you just can’t leave her.” Sometimes helping patients meant sacrificing personal well-being as in the case of one public health nurse working in a seaside community:

One day the boat machine failed while we were two people in it just me and the captain. On our way back from the hospital so patient was at hospital. Boat was moving to the deep sea, I said my God my children will not [find] my dead body.

Participants rationalized their motivation to overcome the difficult work environment similar to this nurse midwife:

…even if I decide that I’m leaving them alone, or say I get angry and stop attending patients, citizens will suffer. It could be that he or she is my neighbor or my relative…

Additional coping mechanisms attributed to influence MLPs to remain in their posts included hope for a better future, religious faith, and support networks comprising spouses, relatives, and friends. Some felt other facilities were similar to their own; it was pointless to uproot life in one area to face similar difficulties in another.

##### Theme II: There is this issue of salaries

Salaries were a major source of dissatisfaction among MLPs. Late salaries, low salaries, partial payment of salaries, and superficial understanding of salary structures were the dominant grievances. A CO interviewed in October 2008 reported that “from May we have not been paid.” Members of CHMTs were said to promise but not compensate personnel for overtime hours worked or reimburse personal funds spent on patient-related activities, such as hiring a car to transport patient to higher levels of care when an ambulance was not available. A nurse assistant reported that “you are just told to fill in the forms but you don’t get paid.” Attempts to resolve salary discrepancies through administrative channels were challenging due to expenses accrued during repeated visits to district headquarters, apathetic response by officials, and inability to fully advocate for oneself. Further aggravating MLPs was the perception that members of CHMTs were exempt from salary predicaments. One nurse midwife explained:

… those seniors [in authority], even if he went [to district authorities] last week, this week he will just be paid. But for those who have been waiting for six months we get told that there’s no money. But for him, there is no problem because he is the boss …

##### Theme III: The working tools are not enough, and there are few workers

MLPs reported severe personnel shortages. One nurse described the impact:

…there are few workers who have been trained here so you find that patient care is not done to an expected standard because there are few trained workers.

Commensurate with inadequate personnel were longer work hours, a heavier workload, and greater patient dissatisfaction due to long wait times. Poor outcomes occurred when precious moments during medical emergencies were wasted locating medications and supplies instead of administering life-saving interventions as in the instance reported by a nurse assistant:

…I found this woman who has just given birth in a very bad situation. So I had to call the doctor and the doctor told me to put her a drip but there was no drip so I had to rush back to the doctor to take [the drip] and I did but when I got back she was even worse. So I had to come to call the doctor but bad enough is when we got back to realize that she was already dead.

MLPs reported lack of proper protective barriers when their work exposed them to potentially infectious bodily fluids, frequent stock outs of essential medications, unreliable electricity, and inadequate water supply. Patients and family members verbalized their dissatisfaction with poor quality of care towards MLPs as one registered nurse stated “…they do complain a lot and sometimes they insult you with harsh words.”

##### Theme IV: I need to upgrade myself, but I cannot

For many respondents, opportunities for both in-service training and advanced professional qualifications were out of reach due to unfair selection criteria applied by members of CHMTs and disqualification based on tenure and education requirements. MLPs were demoralized when members of CHMTs continually selected the same personnel to attend in-service training. Inadequate staffing prevented some MLPs from exploiting available training opportunities to avoid leaving health facilities unattended. Short-term training workshops are highly desired opportunities to obtain new knowledge, earn extra income from stipends, travel away from home, and interact with other attendees. MLPs who were not selected reported their personal and professional growth was stunted. Exposure to poor living conditions constantly reminded MLPs of their inability to advance themselves according to this nurse midwife.

I stayed there a second village from here. There was a day when I was called at 3 am in the midnight to come to attend a pregnant woman who had failed to give birth. I came with a kerosene lamp from there in the night… I did not have a torch…

Sometimes low morale caused tension among MLPs leading to negative interpersonal interactions. One nurse midwife reported:

…You are working in a situation with anger and hatred. Our intention is to save people’s lives but not to build anger.

##### Theme V: How we can team up in order to reduce these hardships in the health sector

Emerging from MLP narratives was the view that they are essential partners in the endeavor for better health outcomes; through collaboration with the government, seemingly intractable health system problems could be overcome. Using interviews to air concerns, one CO asked that policies reflect the true reality of clinical practice “now we beg the government to think about us.” A public health nurse suggested:

…the research you are conducting. We would request that if it is possible people in the ministry should be involved directly. We know that these are the people who make policies… They would come directly and ask us questions and give them our views. In this way I believe that the policies which they make would be better.

The role of government as a reservoir and distributor of technical, fiscal, and human resources influenced the preference for public sector jobs, which are seen to confer greater job security than in the private sector. A CO stated “there are other benefits which are not offered in private facilities.” Pensions are one such benefit.

#### Members of CHMTs

Narratives drawn from interviews of 37 members of CHMTs were analyzed following the same procedure used to sort and compress into categorical data obtained from the MLPs resulting in the six themes presented here.

##### Theme I: We have a shortage in all the cadres

A shortage in all the cadres was acknowledged by all members of CHMTs. Interviewees reported that UAP are more readily available than cadres with higher levels of training. One district health secretary summarized the situation as follows: “we have few health workers while we have great demand.” Recruitment and retention of personnel, particularly for posts in rural areas, was challenging for members of CHMTs. Factors attributed to deter health workers from rural areas include poor infrastructure, traditional housing not connected to the electrical grid, ill-equipped health facilities, low and late salaries, family commitments favoring urban residence, and socio-cultural biases against unmarried personnel.

##### Theme II: There should be a lot of training

According to interviewees, the push to enhance maternal child health services in Tanzania has driven up demand for additional training while leading to an increase in workshops and seminars offered for personnel to gain new knowledge and update skills. Members of CHMTs pointed to personnel shortages as a major factor known to limit the number of health workers who can take advantage of available opportunities. One district health secretary put it this way: “…if the station has one person and that person needs to go for training, this is a problem.” Cadres reported to have the most access to in-service training were COs, nurses, and midwives. We learned that sometimes health workers were taught skills that could not be implemented in their facilities due to lack of resources or personnel were assigned to areas where newly acquired training was not relevant or useful. A district health secretary told us:

…but after she returns from the malaria course you find she goes to do something else not related to malaria. So after a short time those learned skills starts to disappear due to issue of not using them.

##### Theme III: Feedback is provided, and good workers are rewarded

The CHMT interviews mentioned May Day (International Workers’ Day) as an annual event when special gifts are awarded in recognition of exemplary performance:

Of course for the best performance there is a gift. For example this year during May Day we gave a gift to one of our workers… She was the best workers. So there is something like that which motivates people.

Members of CHMTs expressed the view that they communicated expected performance standards to MLPs through the published Health Management Information System, also known by its Swahili acronym, MTUHA. A district medical officer described the process:

…Then there is a book MTUHA No. 2 in which we write and after the supervision we write what we did, we write the date, what we found and the directives we gave. So next time you to visit again you just open your book and see if improvements are done…

In addition to MTUHA, reinforcement of performance standards was reported to occur via various media, including morning staff meetings and during training workshops and seminars.

##### Theme IV: The correlation between workload and workers is that the work is too much compared to number of health workers

In their interviews, members of CHMTs revealed that procedures to match workload to staffing reported in their surveys were impossible to achieve in reality due to severe personnel shortages. One reproductive and child health coordinator reported that:

…you will find one health provider works in antenatal, to provide vaccination, weighing children, providing counseling to mother with children, providing counseling to mothers to come for family planning. One person will provide all these services. Still there are mothers who want to give birth. Therefore you will find that the workload is bigger than workers who are there.

##### Theme V: We are using OPRAS (Open Performance Review and Appraisal System)

Interviewees conveyed the integration of the OPRAS, a formal performance assessment tool and checklist, into the supervision process utilized by members of CHMTs. The degree of objectivity introduced through OPRAS demanded accountability from MLPs, which appealed to members of CHMTs. One reproductive and child health coordinator stated:

…That form is supposed to be filled with year aims so every worker is supposed to work accordingly to this form. So the head of department is supposed to make follow up if those aims are fulfilled and if not there should be reasons why so.

Some members of CHMTs reported that operationalizing OPRAS was problematic. A complete grasp of the performance metrics and the rationale for selecting specific measures still eluded some members of CHMTs. Scheduled supervision visits were postponed on occasion for reasons such as lack of transportation and instances when members of CHMTs were compelled to abandon their supervisory duties and assume the clinician role to relieve personnel burdened by overwhelming patient volume.

##### Theme VI: We have a wide degree of autonomy

With regard to participation of members of CHMTs in matters related to HRH in their districts, interviewees spoke of playing a largely advisory role. Final policy decisions were said to rest with the district executive director but based on real-time intelligence gathered by members of CHMTs concerning the HRH situation on the ground. One district medical officer explained:

…As I said earlier director is the one who is responsible for employing and firing or punishing. As heads of department we can only advice or suggest therefore we must advise our director. So we are advising but we cannot make decision on human resource.

### Summary of qualitative and quantitative results

Our analysis revealed that HRH management is implemented unevenly across the representative sample of 48 districts we examined. We discovered areas in which responses from members of CHMTs and MLPs were concordant, for example, personnel shortages were endorsed by all. In other areas, discordant perspectives were apparent. A summary of qualitative and quantitative results is presented in Table 5.

**Table 5:** Summary of quantitative and qualitative results

HRH management practice	Concordance or discordance^a^	Summary of quantitative and qualitative results
Staffing	Concordant	Members of CHMTs and MLPs agreed that personnel shortages were persistent across all districts and at every facility type.
Appraisal	Concordant	Members of CHMTs reported written supervision requirements were available for each cadre in most districts and more than half of MLPs encountered prearranged formal supervision meetings, which they perceived as adequate.
Workload	Discordant	Members of CHMTs reported availability of procedures to match workload to staffing in majority of districts but most MLPs reported routinely worked unscheduled hours due to personnel shortages and high patient volumes.
Training	Discordant	Members of CHMTs reported selecting MLPs for in-service training based on performance evaluations and training needs, but MLPs reported selection was at the discretion of members of CHMTs using unspecified criteria.
Rewards and recognition	Discordant	Members of CHMTs reported almost all districts reward personnel for performance, but majority of MLPs perceived the rewards to be lacking and unfair.
Participation	Both	Members of CHMTs reported moderate to high levels of autonomy in most HRH management functions, but MLPs were divided on the extent to which members of CHMTs took into account the reality of the practice environment in their decision-making.
Communication	Both	Members of CHMTs reported written standards of supervision were available in most districts and majority of MLPs endorsed possessing a written job description but almost half were routinely asked to perform duties not in their job description.

^a^Concordance or discordance between CHMT and MLP survey and interview responses

## Discussion

The fragmented HRH management and coexistent poor practice environments in our findings reflect the broader context in which important gaps in implementation of Tanzania’s national HRH strategic plan set the stage for lapses at the district level [22]. Despite national policy aimed at reducing the deficit of health workers, the personnel vacancy rate decreased by only 8.1 % between 2006 and 2013 (64.5 % compared to 56.4 %) [22, 53]. With such high personnel vacancy rates, members of CHMTs cannot realistically expect to meet staffing guidelines and MLPs bear the consequences of severe shortages, including burdensome workloads. Similarly, health sector expenditure has remained approximately 10 % of the national budget since the early 2000s, which is below the 15 % recommended for signatories of the Abuja Declaration [22, 53, 54]. The allotment of limited funds in turn imposes budgetary constraints on HRH management activities at all levels of the health system [22, 53]. Viewed through the lens of the basic strategic HR management component model (Fig. 2), misalignment between HRH strategy and actual implementation at the national level means that at the district level HRH management activities can only be partially executed.

According to the Ministry of Health and Social Welfare (MoHSW), key national HRH targets are missed partly because personnel issues are ranked low in priority and also due to inability to effectively appeal for necessary resources from crucial government partners, such as the Ministry of Finance [53]. Difficulty in recruiting personnel reported by members of CHMTs is a prime illustration that missing important national HRH targets, such as budgets, erects an impediment to full implementation of HRH management responsibilities at the district level. In Tanzania, one in three health workers fails to report to their assigned posts [53]. Of the two thirds who reported to post, 13 %—or about 400 of the 3000 personnel who received work permits and turned up to their stations in recent years—left because of delays in remuneration [53]. Prioritizing compensation of health personnel within existing budget allocations is one measure that can abate avertible attrition of the proportion of workers leaving due to salary delays. Establishing and strengthening vital relationships between officials in the Ministries of Health and Finance is an essential first step towards developing a unified personnel remuneration policy.

Besides circumstances arising at the national level, members of CHMTs also contend with their mandate within the decentralized health system in Tanzania, which is to engage community members and nongovernmental partners in setting and achieving district health goals [21]. For meaningful intra- and inter-sectoral collaboration to occur with key stakeholders, members of CHMTs require an additional skillset beyond the conventional clinical know-how; one that is more akin to business operation managers. The CHMT role involves logistical management of limited resources, including personnel and consumable medical products, while striving to meet quality and cost-effectiveness goals. With such complex portfolios to oversee, members of CHMTs would likely benefit from current thinking in operations engineering—the science of optimizing organizational processes to enhance performance—that is increasingly commonplace in the health service industry [55, 56]. Yet, many lack the training to exploit this knowledge. Of the 37 members of CHMTs in our sample, all reported a clinical background but only two possessed postgraduate degrees in public health and administration. Higher education is one conduit through which members of CHMTs can acquire necessary operations competencies [57, 58]. An option for Tanzania to consider is a package of scholarship programs and tuition reimbursement schemes to support graduate education in health care administration [59, 60]. The second approach to instill operations competencies involves supplementing didactic instruction with mentorship for members of CHMTs [57, 59, 60]. To counter existing shortages of qualified and experienced HRH managers, a mentoring program can be arranged with partners from the academic community and private sector [57, 60].

The case for building the capacity of members of CHMTs to create positive practice environments in which MLPs deliver EmOC is grounded in evidence that patients can suffer harmful consequences, including death, when frontline workers are denied proper organizational support. Although the series of seminal studies we report here were conducted in Europe and the USA, their implications are relevant even in low-resource settings. Aiken and colleagues demonstrated that hospital and nursing characteristics, such as workload, educational achievement, and skill mix, had a significant effect on the odds of patient mortality and failure-to-rescue (defined as the inability to prevent a hospitalized patient’s death in the event of a nosocomial complication) [61–65]. In 168 Pennsylvania (USA) hospitals, each additional patient assigned to a nurse was found to increase the likelihood of dying within 30 days of admission by 7 %, the odds of failure-to-rescue by 7 %, and nurse burnout by 23 % [65]. In 12 European countries, more than 30 000 nurses responding to the RN4CAST survey reported leaving patient care tasks undone due to overwhelming workloads and time constraints when nurse-to-patient ratios were between 1:5.4 and 1:13 [61]. The problem with poor practice environments is that they allow unfavorable configurations of care to persist; in Tanzanian facilities, nurse-to-patient ratios as high as 1:77 have been reported [66]. Under such conditions, the attention of personnel is diverted away from important patient care activities so adverse outcomes cannot be prevented.

In our study, both members of CHMTs and MLPs indicated various instances when obstacles in their practice environments threatened or even overshadowed patient care. The lone nurse assistant preoccupied with searching for intravenous fluids instead of administering emergency aid to a critically ill postpartum patient or MLPs taking time off from work responsibilities to seek resolution of salary discrepancies were some emerging themes. At the same time, the capacity of members of CHMTs to address challenges encountered by MLPs in their practice environments was undermined by budget deficits and bureaucratic red tape. These factors prevented members of CHMTs from completing scheduled supervisory visits, relieving payroll bottlenecks that lead to personnel attrition, and meeting staffing guidelines.

Regrettably, narratives depicting poor practice environments have been widely reported across a range of clinical settings in low-income countries and are not unique to Tanzania. Well documented in the global health literature are accounts of personnel dissatisfaction with elements of their practice environments, for example, poor pay in Lebanon [67] and burdensome workloads in Uganda [68] and South Africa [69]. But in Ghana, health professionals who are actively in practice [70] and preservice trainees [71] alike have signaled that presence of supportive HRH management could sway their preferences to favor even rural job postings that are typically less desirable and under-resourced than their urban counterparts. Still to emerge from the available literature is a framework to guide HRH managers in low-income countries on the best way to create positive practice environments for frontline personnel. Existing frameworks, such as the HRH Action Framework [72] and the WHO Working Lifespan Approach, are useful blueprints for managers to plan their HRH strategy. But these frameworks emphasize hierarchical management oversight, which is characteristic of bureaucratic models of work organization [35]. According to Lake [35], professional models of work organization are preferable over bureaucratic models in clinical settings because they emphasize greater presence of qualified staff and mechanisms to support timely and appropriate responses by providers to patient care issues [35]. If the Tanzania context we examined was placed on a spectrum between professional and bureaucratic models of work organization, it would fall closer to the bureaucratic model.

Although interest in some elements of professional models of work organization has been reported in low-income countries, there is no evidence that they have been fully implemented in these regions [73]. Findings from high-income countries show that facilities that have adopted professional models of work organization report optimal practice environments, attract and retain more qualified staff, and produce better patient outcomes. In four US states accounting for more than 20 % of annual hospitalizations nationwide, nurses in Magnet®-designated facilities, accredited with the international hallmark of nursing excellence, reported better work environments than their non-Magnet® counterparts in the same states based on the Practice Environment Scale of the Nursing Work Index (PES-NWI) [74]. Magnet® facilities reported higher proportions of baccalaureate-prepared and specialty-certified nurses than non-Magnet® facilities [74]. In addition, Magnet® facilities had 14 % lower odds of surgical mortality and 12 % lower odds of failure-to-rescue than non-Magnet® facilities [74]. Therefore, leveraging HRH management by members of CHMTs to advance towards a professional model of work organization can be a strategy to meet national maternal health goals. Even when multiple pressing health needs are competing for the same limited resources, better patient outcomes attained in optimal practice environments assert their utility.

Our findings point to four additional target areas as policymakers in Tanzania take incremental steps to improve practice environments encountered by MLPs. First, HRH policy dictates that personnel are entitled to various allowances as inducements to complement their base salary, but these incentives are awarded unevenly [53, 75–77]. Unremitted allowances for uniforms and for overtime hours were a source of dissatisfaction for MLPs in our study and confirm similar findings reported previously [75]. Salary top-ups are attractive benefits for public sector employees, like 88 % of MLPs in our sample, because public sector wages are still not competitive in the current labor market despite recent reforms that saw health sector remuneration increase by more than 100 % between 2005 and 2009 [75, 76]. Ensuring allowances due to MLPs are disbursed regularly and on time would exert additional pressure on an overstretched budget but lessen MLP dissatisfaction. Secondly, inequities in accessing opportunities for continuing professional education and work assignments not properly aligned with newly acquired knowledge are commonplace. According to the MoHSW, such training has not been integrated into the career trajectory of MLPs and career advancement is based on years of service rather than professional development [53]. New avenues for expanding access to continuous education for all MLPs should be explored. Distance learning modules are one option that can potentially be delivered through electronic platforms where available. Thirdly, although OPRAS, the personnel performance evaluation tool, was introduced in 2004 and still being rolled out in 2008, some members of CHMTs in our sample and in prior reports [53, 78] have expressed a lack of proficiency. Unfamiliarity with OPRAS is further compounded by incomplete primary data collection at the facility level when MLPs lack expertise or time to fill in the forms [77, 78]. In such instances, some members of CHMTs may be ill-prepared to assist MLPs overcome obstacles to documentation. As a result, OPRAS is used inconsistently and its full power as a performance improvement tool is not realized. Training and refresher programs should be offered so that members of CHMTs can fully and uniformly utilize OPRAS. Lastly, some MLPs in our sample reported that procedures followed by members of CHMTs to resolve salary discrepancies lacked transparency. These MLPs perceived that salary discrepancies were resolved faster for members of CHMTs who also exhibited public displays of wealth, such as better housing and motor vehicles. While members of CHMTs were not asked and did not speak about their own salary concerns, there have been reports of dominant members of CHMTs deviating from approved budgets and allocating funds to programs other than those identified as priority areas [21]. An independent system to log and investigate MLP grievances, as well as to oversee CHMT compliance, is one mechanism to safeguard against unfair and unethical HRH management practices.

Restructuring HRH management requires upfront fiscal investments and time dedicated to installing related infrastructure and processes. We did not find any studies conducted in low-income countries to estimate costs of implementing comprehensive HRH management reforms. But, we can extrapolate from US facilities fulfilling all of the arduous requirements of the Magnet® model mentioned earlier. Operating costs associated with delivery of inpatient care in these facilities increased by 2.5 % on average and they reached their break-even point within 2–3 years [79]. The financial outlay to implement the Magnet® model was offset by savings gained due to lower personnel turnover and fewer occupational injuries as well as decreased patient mortality and morbidity [80]. Although additional expenditure on HRH management would be expected to stretch already over-committed resources at the outset, it is possible costs will decline as programs mature. Another consideration for low-income countries contemplating investment in HRH management restructuring programs is the role of foreign donors in determining the health agenda of recipient countries [81]. In 2013, Tanzania received more than US$ 1.1 billion in donor aid for health, which was the fourth highest amount worldwide [82]. Majority of donor aid for health is designated for target diseases rather than health system-strengthening initiatives [82]. Within a context in which managers are inclined to prioritize meeting goals set by donors, Dovlo [81] proposes that incentives aimed at enhancing managerial performance should be based on accomplishment of goals relevant to the local setting.

Taken together, qualitative and quantitative data gathered from members of CHMTs and MLPs revealed crucial gaps in HRH management practices implemented in Tanzanian facilities that deliver EmOC. Our analysis found a dearth of empirical evidence generated in low-income countries that can be used to support a package of interventions to strengthen the capacity of members of CHMTs, and such studies are urgently needed. In the absence of pertinent exemplars from low-income countries, we were compelled to rely on findings from studies carried out in high-income countries. Additional research should be conducted to test the effectiveness of recommended interventions aimed at enhancing CHMT operation competencies and improving their familiarity with OPRAS. Effects of interventions to bolster the capacity of members of CHMTs can be compared against quality of practice environments reported by MLPs and measured at distinct time intervals utilizing validated tools, such as the HPWI. Quality of practice environments can also be compared against MLP-sensitive patient outcomes, such as morbidity, mortality, and satisfaction.

It is important to note that MLPs participating in the critical incident interview were asked to relay only *negative* aspects of the practice environment attributed to induce intention to leave. Concerns that comparing negative MLP perspectives to findings from members of CHMTs may have introduced a bias were dispelled for two reasons. First, the 83 MLPs interviewed were a subset of the 837 MLPs who completed the Provider Survey. As a result, we were able to ascertain that themes elicited from the critical incident interview narratives resembled responses made by the larger pool of participants responding to the Provider Survey. Secondly, themes derived from the critical incident interviews overwhelmingly identified deficient HRH management practices as a major source of their dissatisfaction. In fact, despite being asked to speak about only negative events, MLPs voluntarily described positive factors that enabled them to cope with their challenging work environments, such as commitment to the citizens of Tanzania, strong support networks, their own resilience, faith, and hope for the future.

The mixed-method approach utilized in this study was particularly valuable in sorting through the quantitative and qualitative HSSE data to form a unified profile of the state of HRH management practices utilized by members of CHMTs to deploy and manage MLPs. Interview narratives were useful in expanding the meaning of responses provided in the survey data and vice versa. Responses from members of CHMTs either corroborated or contradicted those given by MLPs to generate deeper insight on the HRH management practice reality. Lastly, our study demonstrated the utility of reaching across disciplinary divides for a framework to understand complex phenomena in public health. Wright, Dunford, and Snell’s basic SHRM component model [40, 41] is typically applied in the commerce sector and provided a useful lens through which HRH management practices in Tanzania could be viewed.

### Study limitations

This study was subject to shortcomings associated with self-reports made by members of CHMTs and MLPs. Either group may have attempted to exaggerate positive or negative aspects of the practice environment in order to maintain social desirability. However, the triangulation of data sources and use of quantitative and qualitative data in tandem to inform the research question allowed our findings to converge on the true state of HRH management practice.

The purposeful sampling strategy used in this study constitutes a nonrandom sampling method, which limits generalizations that can be made from the quantitative arm of the study [83]. However, in keeping with the tradition of mixed-method research design, the quantitative findings can only be considered in concert with results from the qualitative arm [43]. The purpose of the quantitative data in this case was to strengthen the qualitative arm of the study by expanding, corroborating, and embellishing the themes elicited from interview narratives [43].

Missing data posed a potential problem during the quantitative analysis phase because it was not possible to revisit participants to obtain missing data or clarify responses. Less than 10 % of the data in each variable we analyzed was missing, and missing variables were scattered randomly across participants. Therefore, cases were not excluded from the analysis based on missing data [84]. Members of the research team with in-depth knowledge of the health services context in Tanzania engaged in peer-debriefing exercises to discuss emerging inferences.

## Conclusions

Our study sheds light on some HRH management practices that are detrimental to practice environments encountered by MLPs in Tanzania. But far from placing responsibility solely on members of CHMTs, our findings affirm the need for additional support in their HRH management role. Additional research conducted in low-income countries is necessary to outline the best way to strengthen the capacity of HRH managers to create positive practice environments for MLPs to deliver consistently high-quality maternal care.

## Abbreviations

AMO, assistant medical officer; CHMT, council health management team; CO, clinical officer; EmOC, emergency obstetric care; HPWI, Healthcare Providers Work Index; HRH, human resources for health; HSSE, Health Systems Strengthening for Equity: The Power and Potential of Mid-Level Providers; MDGs, Millennium Development Goals; MLPs, mid-level providers; OPRAS, Open Performance Review and Appraisal System; SHRM, strategic human resources management; UAP, unlicensed assistive personnel

## Footnotes

1 The International Collaborating Partners for the Positive Practice Environments Campaign include the International Council of Nurses, the International Pharmaceutical Federation, Worldwide Dental Federation, World Medical Association, Global Health Workforce Alliance, International Hospital Federation, and the World Confederation for Physical Therapy.

2 Council health management teams comprise the district medical officer (chairperson), district nursing officer, district laboratory technician, district health officer, district pharmacist, district dental officer, and district health secretary. Other members may include the reproductive and child health coordinator, tuberculosis and leprosy coordinator, malaria coordinator, HIV/AIDS coordinator, and cold chair operator.

3 The signal functions used to identify basic EmOC include (1) administration of parenteral antibiotics, (2) administration of uterotonic drugs, (3) administration of parenteral anticonvulsants for preeclampsia and eclampsia, (4) manual removal of the placenta, (5) removal of retained products of conception by manual vacuum extraction and dilation and curettage, (6) performing assisted vaginal delivery using vacuum and forceps, and (7) performing basic neonatal resuscitation with a bag and mask. The signal functions used to identify comprehensive EmOC include the previously mentioned seven functions plus (8) performing surgery, such as a cesarean section, and (9) performing a blood transfusion.
